# Genotyping microarray: Mutation screening in Spanish families with autosomal dominant retinitis pigmentosa

**Published:** 2012-06-05

**Authors:** Fiona Blanco-Kelly, María García-Hoyos, Marta Cortón, Almudena Ávila-Fernández, Rosa Riveiro-Álvarez, Ascensión Giménez, Inma Hernan, Miguel Carballo, Carmen Ayuso

**Affiliations:** 1 Servicio de Genética, IIS Fundación Jiménez Díaz, Madrid. Centro de Investigación Biomédica en Red de Enfermedades Raras (CIBERER), ISCIII, Madrid, Spain; 2 Unidad de Genética Molecular, Hospital de Terrassa, Terrassa, Barcelona, Spain

## Abstract

**Purpose:**

Presently, 22 genes have been described in association with autosomal dominant retinitis pigmentosa (adRP); however, they explain only 50% of all cases, making genetic diagnosis of this disease difficult and costly. The aim of this study was to evaluate a specific genotyping microarray for its application to the molecular diagnosis of adRP in Spanish patients.

**Methods:**

We analyzed 139 unrelated Spanish families with adRP. Samples were studied by using a genotyping microarray (adRP). All mutations found were further confirmed with automatic sequencing. Rhodopsin (*RHO*) sequencing was performed in all negative samples for the genotyping microarray.

**Results:**

The adRP genotyping microarray detected the mutation associated with the disease in 20 of the 139 families with adRP. As in other populations, *RHO* was found to be the most frequently mutated gene in these families (7.9% of the microarray genotyped families). The rate of false positives (microarray results not confirmed with sequencing) and false negatives (mutations in *RHO* detected with sequencing but not with the genotyping microarray) were established, and high levels of analytical sensitivity (95%) and specificity (100%) were found. Diagnostic accuracy was 15.1%.

**Conclusions:**

The adRP genotyping microarray is a quick, cost-efficient first step in the molecular diagnosis of Spanish patients with adRP.

## Introduction

Retinitis pigmentosa (RP, OMIM 268000) is the most common form of inherited retinopathy, with a prevalence of approximately one in 4,000 [1]. RP is a group of clinically and genetically heterogeneous retinal degenerative diseases characterized by progressive loss of photoreceptors and pigment deposits predominantly in the peripheral retina and by a relative sparing of the central retina. The diagnostic criteria for RP were established by Marmor [2-5]. To date, 56 genes have been associated with non-syndromic RP (RetNet), and all modes of inheritance have been described in this disease (autosomal dominant, autosomal recessive, X linked, and in rare cases, digenic) [6].

Autosomal dominant RP (adRP) accounts for approximately 15% of Spanish families with RP [7,8]. The large number of genes involved in adRP complicates genetic analysis in these patients. To date, 22 genes have been associated with adRP (RetNet):

Bestrophin (*BEST1*, 11q12.3), carbonic anhydrase IV (*CA4*, 17q23), cone-rod homeobox (*CRX*, 19q13.3), fascin homolog 2 (*FSCN2*, 17q25), guanylate cyclase activator 1B (*GUCA1B*, 6p21.1), inosine 5′-monophosphate dehydrogenase 1(*IMPDH1*, 7q32.1), kelch-like 7 (*Drosophila*; *KLHL7*, 7p15.3), nuclear receptor subfamily 2, group E, member 3 (*NR2E3*, 15q22.32), neural retina leucine zipper (*NRL*, 14q11.2), PRP3 pre-mRNA processing factor 3 homolog (*S. cerevisiae*; *PRPF3*, 1q21.1), PRP6 pre-mRNA processing factor 6 homolog (*S. cerevisiae*; *PRPF6*, 20q13.33), PRP8 pre-mRNA processing factor 8 homolog (*S. cerevisiae*; *PRPF8*,17q13.3), PRP31 pre-mRNA processing factor 31 homolog (*S. cerevisiae*; *PRPF31*, 19q13.4), peripherin 2 (RDS / PRPH2, 6q21.2), retinol dehydrogenase 12 (all-trans/9-cis/11-cis) (*RDH12*, 14q24.1), rhodopsin (RHO, 3q21–24), retinal outer segment membrane protein 1 (*ROM1*, 11q13), retinitis pigmentosa 1 (*RP1*, 8q11.3), retinitis pigmentosa 9 (*RP9*, 7p14.3), sema domain, immunoglobulin domain (Ig), transmembrane domain (TM) and short cytoplasmic domain, (semaphorin) 4A (*SEMA4A*, 1q22), small nuclear ribonucleoprotein 200 kDa (U5) (*SNRNP200*, 2q11.2) and topoisomerase I binding, arginine/serine-rich, E3 ubiquitin protein ligase (*TOPORS*, 9q21.1). Patients with adRP, from informative families, are studied with linkage analysis, or gene-by-gene, according to their established prevalence in the analyzed population. This method is costly, requires substantial human resources, and is time-consuming. However, only around 15%–60% of adRP cases can be explained by mutations in these genes [8-12], and the detection ratio depends on the population studied and the analytical technique [8-12], thus making molecular diagnosis and prognosis more difficult and complex.

The aim of this study was to evaluate a specific adRP genotyping microarray (Asper Biotech, Ltd., Tartu, Estonia) for application to the molecular diagnosis of Spanish families with adRP. We selected and analyzed, using the genotyping microarray, 139 Spanish families with clinical diagnosis of RP as well as pedigree evidence indicating the autosomal dominant mode of inheritance (according to previously established criteria [7,8]).

## Methods

### Patients

We studied the molecular causes of the disease in 139 unrelated Spanish families with adRP recruited from 1990 to May 2011. Seventy-eight of these families had been previously screened for *CRX* (Chromosome 19q13.3), *FSCN2* (17q25), *IMPDH1* (7q32.1), *NRL* (14q11.2), *PRPF3* (1q21.1), *PRPF8* (17q13.3), *PRPF31* (19q13.4), *RDS* (6q21.2), *RHO* (3q21–24), *ROM1* (11q13), and *RP1* (8q11.3), with other molecular methods (denaturing gradient gel electrophoresis and sequencing verification), with negative results, and 61 had had no prior testing.

The adRP diagnosis was based on ophthalmologic examination and pedigree data. We classified our patients as affected by RP according to the following clinical criteria: night blindness, progressive loss of peripheral vision (midperipheral scotoma or ring scotoma), fundus compatible with RP (with altered retinal pigment epithelium, black bone spicule-like pigmentation, pallor of the optic nerve head, and retinal vessel attenuation), and pathologic electroretinogram showing a marked reduction in rod or rod and cone signaling [2-5]. Autosomal dominant inheritance was assessed according to previously established criteria [7,8], either the presence of three or more generations, with both men and women among all affected family members, or at least two affected generations with male-to-male transmission; these requirements reduced the likelihood of including X-linked families in our study.

Informed consent was obtained from all persons involved in the study, and research protocols were approved by the Bioethical Committee, of the IIS-Fundación Jiménez Díaz, in accordance with the tenets of the Declaration of Helsinki.

One affected member of each family was analyzed using the adRP genotyping microarray. Whenever a sequence change was detected and confirmed, the study was extended to the rest of the family.

### Screening for mutations

DNA was extracted from peripheral blood samples collected in EDTA tubes using an automated DNA extractor according to the manufacturer’s instructions (model BioRobot EZ1; Qiagen, Hilden, Germany).

We first used a specific adRP genotyping microarray for mutation screening [13] (ADRP genetest). This genotyping microarray is based on Arrayed Primer Extension (APEX) technology. We used two versions of the array. The first version was used from October 2007 to September 2009, and contained 353 disease-associated sequence variants in 13 adRP genes (*CA4*, *FSCN2*, *IMPDH1*, *NRL*, *PRPF3*, *PRPF31*, *PRPF8*, *RDS, RHO*, *ROM1*, *RP1*, *RP9*, and *CRX*). The second version was used from September 2009 to June 2011, and had 386 disease-associated sequence variants in 16 adRP genes (*NR2E3*, *KLHL7*, and *TOPORS* were added to the previous 13 genes, one mutation for each gene). Direct sequencing, using the BigDye Terminator Cycle Sequencing v3.1 kit (Applied Biosystems, Carlsbad, CA) and analyzing purified sequencing reactions in an ABI PRISM 3730 DNA analyzer *(*Applied Biosystems), was used to confirm the results obtained with the genotyping microarray and to segregate the disease-causing mutations in the families (all mutations detected, segregated with the disease).

Additionally, in uncharacterized families analyzed before 2009, missense mutation p.Gly56Arg in the *NR2E3* gene was analyzed as previously reported [14] until it was introduced in the microarray in September 2009. As *RHO* is the most mutated gene among Spanish patients with adRP [9], analysis of all five exons of the *RHO* gene was performed using intragenic primers, as previously described [15], for all negative array results to detect false negatives.

### Statistical analysis

Specificity and sensitivity calculations were performed using the free software package Epidat 4.0 (Organización Panamericana de la Salud y Consellería de Sanidade de la Xunta de Galicia, 2011). Differences in the detection ratio were analyzed using the χ^2^ test included in Epidat 4.0.

## Results

Using the adRP genotyping microarray, we detected the mutation associated with the disease in 21 of the 139 families with adRP (15.1%). There were no false positives, as all mutations previously detected with the adRP genotyping microarray were confirmed and segregated with sequencing analysis. One false negative, for the p.Glu181Lys mutation in *RHO* (the mutation included in the microarray), was detected after *RHO* sequencing. This gives us an array sensitivity not higher than 95%, as the mutations in other genes (not *RHO*) present in the array were not sequenced on negative samples, and specificity of 100% for the spots present in the microarray.

The microarray detected mutations in ten patients out of the 78 previously studied families (12.8%) and in 10 of the 61 (16.4%) of the families analyzed for the first time. There was no statistical difference in the detection ratio between both groups (p=0.28).

Additionally, previously studied families with negative results underwent analysis of the p.Gly56Arg mutation in *NR2E3*, before it was included in the microarray. The analysis of this mutation allowed us to characterize five more adRP families.

The sequencing of *RHO* in those patients who were negative according to the adRP genotyping microarray testing (119) allowed us to characterize one additional family.

**Figure 1:**
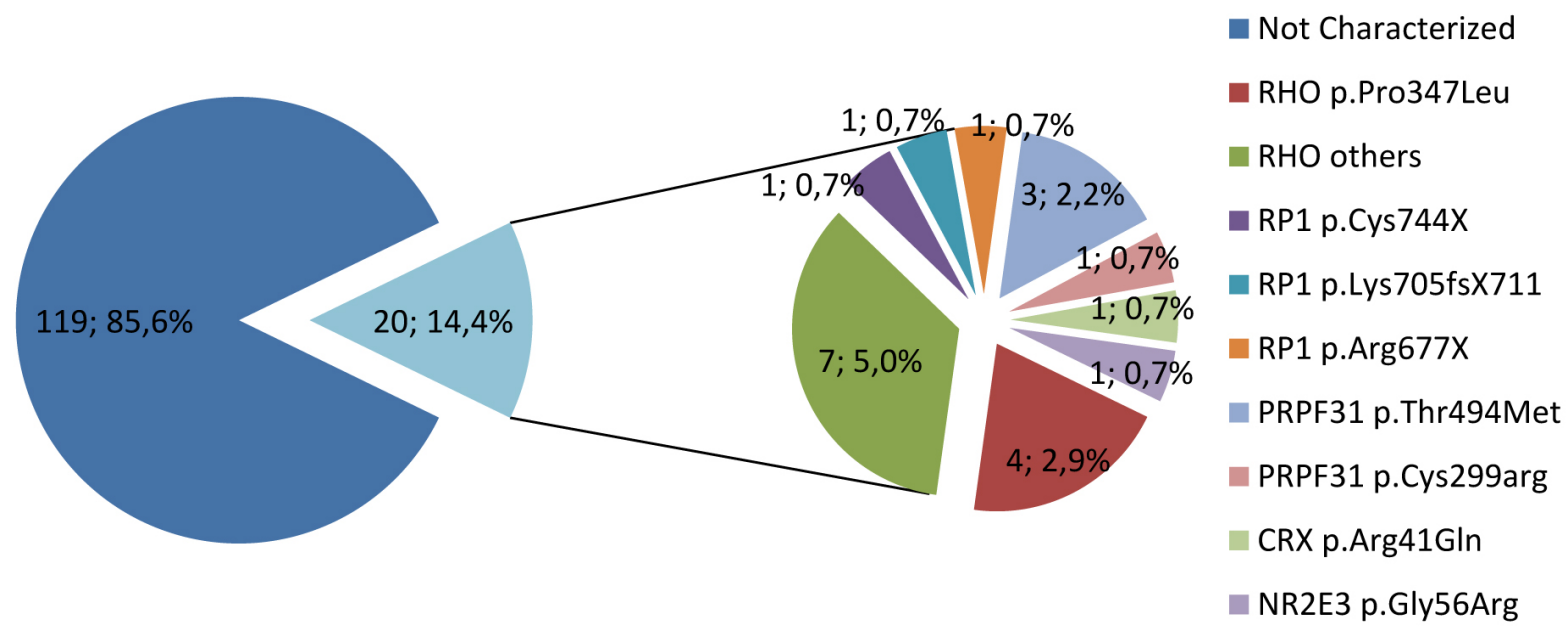
Total number and percentage of mutations detected with microarray genotyping in our cohort of families with autosomal dominant retinitis pigmentosa (20 out of 139).

A total of 26 mutations were associated with adRP in the tested population, 21 of which were detected with genotyping microarray and six with direct sequencing. The genes in which mutations were found are *RHO*, *NR2E3*, *PRPF31*, *RP1*, and *CRX.* Eleven of the families with mutations had a mutation in *RHO*: ten were detected with the microarray and one with direct sequencing. Six families presented with the p.Gly56Arg mutation in *NR2E3* (one detected with the adRP genotyping microarray and five with direct sequencing before the mutation was included in the microarray). The other mutations were all detected with the microarray: four in *PRPF31*, three in *RP1*, and one in *CRX*. Table 1 shows the distribution of the mutations around the adRP genes in the cohort of Spanish families studied. Figure 1 shows the frequency of adRP mutations detected with microarray genotyping in our cohort of adRP families (n=139).

**Table 1:** Mutations detected in the adRP Spanish cohort (n=139)

**Gene**	**Mutation**	**Number of mutated families**
***RHO***		
	p.Gly106Arg	1
	p.Arg135Leu	1
	p.Ala164Glu	1
	p.Pro171Leu	1
	p.Tyr178Cys	1
	p.Glu181Lys	1*
	p.Gly182Ser	1
	p.Asp190Tyr	1
	p.Pro347Leu	4
***RP1***		
	p.Arg677Stop	1
	p.Lys705fsX711	1
	p.Cys744Stop	1
***PRPF31***		
	p.Cys299Arg	1
	p.Thr494Met	3
***CRX***		
	p.Arg41Gln	1
***NR2E3***		
	p.Gly56Arg	1 (microarray genotyping) +
		5 (direct sequencing)
**Total**		20 (microarray genotyping) + 6 (direct sequencing)

*Detected by direct sequencing, microarray genotyping false negative.

## Discussion

Although several studies have been published on the use of this kind of genotyping microarray for molecular characterization of other retinal dystrophies (Stargardt disease [16,17], Leber congenital amaurosis [18,19], Usher syndrome [20,21], and autosomal recessive RP [22]), this is the first study to be published on the sensitivity and specificity of the adRP genotyping microarray for application to molecular diagnosis of patients with adRP.

In this study, we performed a molecular analysis of 139 Spanish families with adRP with the adRP genotyping microarray, and in all the cases, sequencing analysis confirmed the mutation previously detected with the genotyping microarray.

With the subsequent sequencing analysis of the *NR2E3* dominant mutation (in those who were negative for microarray testing), we identified the causative gene in 17.4% of the families studied. In our cohort of patients, we detected 26 different mutations, 13 in more than one family: p.Gly56Arg in the *NR2E3* gene (in six families), p.Pro347Leu in the *RHO* gene (in four families), and p.Thr94Met in *PRPF31* (in three families). In Spanish families, the mutation p.Gly56Arg in the *NR2E3* gene is the most common single mutation detected in our patients with adRP, representing 10% (6/60) of the total of our mutated families (four of these families reside in the same small city in Spain, and a further study to find a common ancestor is under way).

Interestingly, although mutations in *RHO* are the most commonly associated cause of adRP according to other analyses (16% [23] to 26% [9,24]), in our study *RHO* was responsible for 7.9% of the total families studied. This could be because not all reported mutations for *RHO* are included in the microarray. In fact, in all of our families (data not shown) *RHO* accounts for 53.3% of all mutated families, 32 out of 60, from which 21 were diagnosed with denaturing gradient gel electrophoresis with sequencing confirmation [25,26] before the introduction of the adRP genotyping microarray (20 of those mutations are actually included in the array), and 18.6% of all the families with adRP (32 out of 172). Fifty-seven of the 60 mutations found in *RHO* are present in the adRP genotyping microarray; therefore, analyzing the diagnostic accuracy of a protocol for adRP including genotyping microarray followed by *RHO* sequencing would be interesting, as *RHO* is the most mutated gene among Spanish patients with adRP [9] and a considerable percentage of mutations are not covered by the array (5%).

In other populations, mutations in known adRP genes are estimated to account for at least 50%–60% of adRP cases [9-12], although recurrent mutations (p.Pro23His in *RHO*, c.828+3A>T in *RDS*, and p.Leu762fsX and p.Arg677X777 in *RP1*) account for around 35% of all cases, and novel mutations are found in the others [12]. In our study, only three mutations are recurrent, accounting for 65% (13/20) of the total mutations detected with the microarray.

Our results show the adRP genotyping microarray is useful as the first step in molecular diagnosis of Spanish families with adRP. This technology provided a quick and cost-effective method for performing the previously described adRP-causative mutation analysis. The diagnostic accuracy of adRP genotyping microarray in this study was 14.4%, but the most recent version of the microarray also contains the mutation p.Gly56Arg in the *NR2E3* gene, which, in this cohort, would have allowed diagnosis in 18% of the families (25 out of 139). The adRP genotyping microarray for genetic diagnosis of families with autosomal dominant RP could be used as the first step to diagnose them, though the microarray must be updated periodically due to the discovery of new genes and mutations responsible for adRP. The microarray could also be used as the second step for previously uncharacterized families. The adRP genotyping microarray is an efficient approach for adRP diagnosis, although families uncharacterized with the adRP genotyping microarray must undergo complementary analysis to continue research to identify new disease-causing mutations in the genes associated with adRP.

Identifying the underlying disease-causing mutation in families with adRP is an essential step in diagnosis and genetic counseling. Moreover, faster detection of the mutations of this disease will allow patients earlier access to gene-based therapy when it is developed.
